# HLA Class I and II Expression in Oropharyngeal Squamous Cell Carcinoma in Relation to Tumor HPV Status and Clinical Outcome

**DOI:** 10.1371/journal.pone.0077025

**Published:** 2013-10-10

**Authors:** Anders Näsman, Emilia Andersson, Linda Marklund, Nikolaos Tertipis, Lalle Hammarstedt-Nordenvall, Per Attner, Tommy Nyberg, Giuseppe V. Masucci, Eva Munck-Wikland, Torbjörn Ramqvist, Tina Dalianis

**Affiliations:** 1 Department of Oncology-Pathology, Karolinska Institutet, Stockholm, Sweden; 2 Department of Oto-Rhino-Laryngology, Head and Neck Surgery, Karolinska Institutet, Karolinska University Hospital, Stockholm, Sweden; University of Torino, Italy

## Abstract

HPV-DNA positive (HPV_DNA_+) oropharyngeal squamous cell carcinoma (OSCC) has better clinical outcome than HPV-DNA negative (HPV_DNA_-) OSCC. Current treatment may be unnecessarily extensive for most HPV+ OSCC, but before de-escalation, additional markers are needed together with HPV status to better predict treatment response. Here the influence of HLA class I/HLA class II expression was explored. Pre-treatment biopsies, from 439/484 OSCC patients diagnosed 2000-2009 and treated curatively, were analyzed for HLA I and II expression, p16^INK4a^ and HPV DNA. Absent/weak as compared to high HLA class I intensity correlated to a very favorable disease-free survival (DFS), disease-specific survival (DSS) and overall survival (OS) in HPV_DNA_+ OSCC, both in univariate and multivariate analysis, while HLA class II had no impact. Notably, HPV_DNA_+ OSCC with absent/weak HLA class I responded equally well when treated with induction-chemo-radiotherapy (CRT) or radiotherapy (RT) alone. In patients with HPV_DNA_- OSCC, high HLA class I/class II expression correlated in general to a better clinical outcome. p16^INK4a^ overexpression correlated to a better clinical outcome in HPV_DNA_+ OSCC. Absence of HLA class I intensity in HPV_DNA_+ OSCC suggests a very high survival independent of treatment and could possibly be used clinically to select patients for randomized trials de-escalating therapy.

## Introduction

The incidence of oropharyngeal squamous cell carcinoma (OSCC) is increasing, mainly due to a rise in human papillomavirus (HPV) DNA positive HPV (HPV_DNA_+) OSCC, suggesting an epidemic of viral-induced OSCC[1–4]. This may be of importance for the treatment of OSCC, where tonsillar squamous cell carcinoma (TSCC) and base of tongue squamous carcinoma (BOTSCC) dominate[5], since HPV_DNA_+ tumors have a much better clinical outcome than those that are HPV DNA negative (HPV_DNA_-)[6,7]. More specifically, patients with HPV_DNA_+ tumors have roughly an 80% 5-year disease-specific survival, compared those with HPV_DNA_- tumors, where survival (40%) is similar to that observed in patients with other head and neck squamous cell carcinomas (HNSCC) of similar stages[6,8]. 

The fact that most HNSCC patients present with a poor prognosis has resulted in an intensification of the oncological treatment, resulting in a significant increase in acute and late sequele. All patients with HPV_DNA_+ OSCC may not benefit from intensified treatment, and to decrease the severe side-effects, it has been proposed to reduce treatment for this group. However, since a significant proportion of patients with HPV_DNA_+ OSCC have a poor clinical outcome, additional predictive markers are needed, before introducing a possible de-escalation of treatment[9,10]. 

Extensive data suggest that HPV_DNA_+ OSCC is a different disease-entity from HPV_DNA_- OSCC, and the two should be analyzed separately when searching for additional predictive markers[11]. Furthermore, HPV status can be defined by different methods, e.g. as HPV_DNA_+, or as HPV_DNA_+p16^INK4a^ overexpression or as sometimes by p16^INK4a^ overexpression alone - since p16^INK4^ overexpression is considered a marker of active HPV expressing E7 mRNA[12]. 

In a previous smaller study, we showed that absent/weak HLA class I expression correlated with a very favorable outcome in HPV_DNA_+ TSCC, while the opposite was observed in HPV_DNA_- TSCC[13]. It is possible that HLA class I downregulation was due to that viral E5 and E7 oncoproteins have the potential to interfere with the HLA class I presenting machinery[14–16]. 

In contrast to downregulation of HLA class I expression, HLA class II antigen expression, normally not present in epithelial cells, can be observed in, for instance, cervical cancer[17–19]. Moreover, *in vitro* HLA class II expression on epithelial cells has been shown to enhance tumor-specific immunity by bypassing the classical antigen-presenting cell-mediated pathway[20,21]. Moreover, HLA class II expression can be linked to both better and worse prognoses in a variety of malignancies, but has not been studied in OSCC[22–26]. 

Here, in OSCC, from a large cohort of patients, HLA class I and II expression was analyzed in relation to HPV status and clinical outcome. This extends our previous investigation on the predictive value of HLA class I expression on clinical outcome. 

## Materials and Methods

### Patients, tumor biopsies and treatment

The local cancer registry (>98% complete) was used to identify patients with OSCC (defined as ICD-10 codes: C09, C01.9, C05.1-8 and C10) diagnosed in the County of Stockholm between January 2000 and October 2009 (C09 and C01.9, for tonsillar and base of tongue cancer respectively) and January 2000 and January 2009 (C05 and C10, for OSCC other than tonsillar and base of tongue cancer). Eligibility criteria were presence of available pathologically verified pre-treatment biopsies and curative treatment with RT. Patient records were then evaluated to verify the diagnosis and to collect patient characteristics (Table 1). 

**Table 1:** Characteristics of patients* included in the study and their tumors.

			**HPV positive OSCC patients (N=303)**		**HPV negative OSCC patients (N=136)**		**All OSCC patients (N=439)**		**p value**
**Patient characteristics**			N	%	N	%	N	%	
**Age**	*Mean (years)*		60		63		61		<0.001
	*Median (years)*		59		62		60		
	*Range (years)*		30-90		30-87		30-90		
	*Inter-quartile range (years)*		53-66		56-71		54-67		
**Diagnose**	*malignant neoplasm of the base of tongue (C01.9)*		75	25%	28	21%	103	24%	<0.001
	*malignant neoplasm of the palate (C05.0-9)*		7	2.3%	15	11%	22	5.0%	
	*malignant neoplasm of the tonsil (C09.0-9)*		217	72%	66	49%	283	65%	
	*malignant neoplasm of the oropharynx (C10.0-9)*		4	1.3%	27	20%	31	7.1%	
**Sex**	*female*		80	26%	39	29%	119	27%	0.64
	*male*		223	74%	97	71%	320	73%	
**Tumour differentiation**	*poor*		198	65%	78	57%	276	63%	0.052
	*moderate*		89	29%	45	33%	134	31%	
	*well*		7	2.3%	10	7.4%	17	3.9%	
	*undefined*		9	3.0%	3	2.2%	12	2.7%	
**Tumour size**	*T1*		75	25%	19	14%	94	21%	0.009
	*T2*		110	36%	43	32%	153	35%	
	*T3*		57	19%	40	29%	97	22%	
	*T4*		61	20%	34	25%	95	22%	
**Nodal disease**	*N0*		49	16%	54	40%	103	23%	<0.001
	*N1*		70	23%	17	13%	87	20%	
	*N2a*		47	16%	13	10%	60	14%	
	*N2b*		96	32%	21	15%	117	27%	
	*N2c*		29	10%	22	16%	51	12%	
	*N3*		10	3.3%	8	5.9%	18	4.1%	
	*NX*		2	0.66%	1	0.74%	3	0.68%	
**Distant metastasis**	*M0*		297	98.0%	132	97%	429	97.7%	0.17
	*M1*		3	1.0%	0	0%	3	0.68%	
	*MX*		3	1.0%	4	2.9%	7	1.6%	
**Tumour Stage**	*I*		4	1.3%	10	7.4%	14	3.2%	0.009
	*II*		22	7.3%	14	10%	36	8.2%	
	*III*		76	25%	33	24%	109	25%	
	*IVa*		183	60%	68	50%	251	57%	
	*IVb*		15	5.0%	11	8.1%	26	5.9%	
	*IVc*		3	1.0%	0	0.0%	3	0.68%	
**Treatment**	*Induction chemotherapy and radiation*	*conventional*	146	48%	85	63%	231	53%	0.18
		*accelerated*	57	19%	15	11%	72	16%	
	*Radiation*	*conventional*	28	9.2%	9	6.6%	37	8.4%	
		*accelerated*	72	24%	27	20%	99	23%	
**Brachytherapy boost**	*Not administered*		240	79%	102	75.0%	342	78%	0.32
	*Administered*		63	21%	34	25.0%	97	22%	
**Concomittant Cetuximab**	*Not administered*		265	87%	125	92%	390	89%	0.19
	*Administered*		38	13%	11	8.1%	49	11%	
**Smoking**	*Never*		98	32%	14	10%	112	26%	<0.001
	*Former (>15 years ago)*		54	18%	7	5.1%	61	14%	
	*Former (<15 years ago)*		52	17%	13	10%	65	15%	
	*Current upon diagnosis*		99	33%	102	75%	201	46%	
**p16^INK4a^ expression**	*positive*		246	81%	15	11%	261	59%	<0.001
	*negative*		57	19%	121	89%	178	41%	
*Number of patients with OSCC according to the local Cancer Registry, and after reviewing patients' records:						551 patients			
Number of patients excluded, not meeting the incusion criteria, due to:									
	*Patients without pre-treamtent biopsies available*					45 patients			
	*Patients with palliative treatment only*					63 patients			
	*Patients with surgical treatment only*					4 patients			

Treatment was classified as radiotherapy (RT) (up to 68Gy in a conventional or in an accelerated setting) or induction chemo-RT (CRT) (Cisplatin+5Fu with/without Docetaxel – or, as in a smaller number of cases, Cisplatin+Docetaxel+Capecitabine – followed by conventional/accelerated RT). If brachytherapy was added, a total dose up to 78Gy was given. Moreover, some patients also received concomitant Cetuximab treatment (Table 1). Before mid-2007, treatment for patients with regional metastases also included neck dissection. Thereafter, neck dissection was performed only in patients with N2c or N3, and those who had remaining palpable neck nodes after oncological treatment. Smoking data were collected and categorized as: never smoked; stopped >15 years ago; stopped <15 years ago and current smoker (Table 1). 

A written consent was given by the patients for their information to be stored in the hospital database and to be used for research. The study was conducted according to ethical permissions 2005/431-31/4, 2005/1330-32 and 2009/1278-31/4 from the Regional Ethical Committee at Karolinska Institutet.

### HPV DNA analysis

DNA was extracted from 30µm paraffin-embedded pre-treatment biopsy slices, as previously described[2]. Presence of HPV DNA was analyzed using a bead-based multiplex assay on a MagPix instrument (Luminex Corporation), as described elsewhere[27].

### Immunohistochemistry

HLA class I heavy chains were detected using the mouse monoclonal antibodies (mAb) HCA-2 and HC-10, (HCA-2 recognizes most HLA-A and HC-10 most HLA-B and -C heavy chains, with some overlaps) and HLA class II antigens using mAb LGII-612.14 (recognizes HLA-DR –DQ and DP, but not other HLA class II antigens). These antibodies, kind gifts from Dr Soldano Ferrone, University of Pittsburgh, Cancer Institute, PA, USA, have been extensively described elsewhere[28–31]. Expression of p16^INK4a^ was detected using the mAb p16^INKA4a^ (clone: JC8, dilution 1:100, Santa Cruz Biotech, California, U.S.A.).

Staining, with negative and positive controls, was performed as previously described[13] and evaluated blind by two investigators (AN and EA). In the case of disagreement a consensus was made. Fractions of HLA class I and II positive cells were evaluated semi-quantitatively as five grades: 0 (0%), 1 (1-25%), 2 (26-50%), 3 (51-75%), and 4 (76-100%). Staining intensity was also evaluated and scored on a three-tier scale as absent, weak and strong staining[13]. Expression of p16^INK4a^ was scored as positive (strong nuclear staining in >70% cells) or as negative staining. (Figure S1 shows examples of staining for HLA class I and p16^INK4a^).

### Statistical analysis

The Chi square test was used for categorical data and the student t-test to compare mean values. 

Survival was measured in years from the date of diagnosis until a defined event or until 3 years after diagnosis, when patients were censored. An event was defined as death due to any cause (overall survival, OS), death with OSCC present (disease-specific survival, DSS) or recurrence in OSCC (disease-free survival, DFS). Patients who died without a documented OSCC present were considered as a censored observation in DSS and patients who died without a prior recurrence were censored at day 0 in DFS. The Kaplan-Meier estimator was used to estimate DFS, DSS and OS. Differences in survival were tested using the log-rank test. The Cox proportional hazards model was used to calculate the unadjusted and adjusted hazard ratios (HR).

All tests were performed two-sided at the 5% significance level. All calculations were performed using SAS software (ver. 9.3, SAS Institute Inc., Cary, NC, USA).

## Results

### Patients, HPV and tumor characteristics

In total, 551 patients were identified with OSCC, and 439 fulfilled the inclusion criteria e.g. treated with curative intent and with available diagnostic pre-treatment biopsies (Table 1), while 45 patients treated with curative intent without available biopsies were excluded from the analysis (Table S1). 

Altogether, 303/439 (69%) of the OSCC were HPV_DNA_+, with the majority of HPV_DNA_+ cases being represented by TSCC (217/283, 77%) and BOTSCC (75/103, 73%) respectively. Tumors in the soft palate and oropharynx harbored HPV_DNA_ more rarely - 7/22 (32%) and 4/31 (13%) respectively (Table 1). Overexpression of p16^INK4a^ was significantly more frequently observed in HPV_DNA_+ (p<0.001) compared to HPV_DNA_- OSCC. However, when analyzed in the different sub-sites separately, significant correlations between HPV_DNA_ and p16^INK4a^ were only observed in TSCC and BOTSCC (both p<0.001).

Patients with HPV_DNA_+ OSSC, when compared to patients with HPV_DNA_- OSCC, were younger (p<0.001); more likely never to have smoked (p<0.001); presented significantly more frequently with smaller tumors (p=0.009); had greater nodal disease (p<0.001); and had a higher tumor stage (p=0.009) (Table 1).

Treatment modalities were similar for patients with HPV_DNA_+ and HPV_DNA_- OSCC (Table 1).

The 45 patients treated with curative intent who were excluded from the study due to the unavailability of biopsies only differed from the group included in the analysis in terms of treatment, where administration of conventional RT dominated (Table S1).

### HLA class I and II expression and HPV in OSCC

In HPV_DNA_+ OSCC, the fraction and intensity of HLA class I expressing cells were generally lower, and the fraction and intensity of HLA class II expressing cells were higher compared to HPV_DNA_- OSCC (Table 2). 

**Table 2:** HLA class I and II exptression in HPV DNA positive and HPV DNA negative oropharyngeal squamous cell carcinoma patients.

		**HPV_DNA_ positive status**		**HPV_DNA_ negative status**		
		N	%	N	%	p-value^§^
**Intensity of HCA-2 positive cells**	*absent*	101	33%	24	18%	0.001
	*weak*	60	20%	45	33%	
	*strong*	142	47%	67	49%	
**Fraction of HCA-2 positive cells**	*absent*	101	33%	24	18%	0.009
	*1-25%*	33	11%	14	10%	
	*26-50%*	24	8%	16	12%	
	*51-75%*	33	11%	14	10%	
	*76-100%*	112	37%	68	50%	
**Intensity of HC-10 positive cells**	*absent*	60	20%	9	7%	0.001
	*weak*	73	24%	33	24%	
	*strong*	170	56%	94	69%	
**Fraction of HC-10 positive cells**	*absent*	60	20%	9	7%	0.001
	*1-25%*	24	8%	7	5%	
	*26-50%*	16	5%	4	3%	
	*51-75%*	39	13%	15	11%	
	*76-100%*	164	54%	101	74%	
**Intensity of LGII-612.14 positive cells**	*absent*	100	33%	82	60%	<0.001
	*weak*	29	10%	11	8%	
	*strong*	174	57%	43	32%	
**Fraction of LGII-612.14 positive cells**	*absent*	100	33%	82	60%	<0.001
	*1-25%*	26	9%	10	7%	
	*26-50%*	23	8%	7	5%	
	*51-75%*	34	11%	12	9%	
	*76-100%*	120	40%	25	18%	

§ Chi-square test

### HPV and survival in OSCC patients

Patients with HPV_DNA_+ OSCC had a significantly better DFS, DSS and OS than patients with HPV_DNA_- OSCC (p<0.001 by the log-rank test for all three end-points). The 3-year DFS in the HPV_DNA_+ and the HPV_DNA_- groups was 88% (95% CI 84-91) and 66% (95% CI 56-75) respectively. Corresponding numbers in the two groups for DSS were: 88% (95% CI 84-91) and 59% (95% CI 49-67) respectively; and for OS 84% (95% CI 79-88) and 51% (95% CI 42-59) respectively.

In a multivariate analysis, including sex, age, tumor localization and stage, HPV_DNA_+ status was still a highly significant determinant of survival. The unadjusted hazards ratios for DFS were: 0.30 (95% CI 0.19-0.48); for DSS: 0.23 (95% CI 0.15-0.36) and for OS: 0.26 (95% CI 0.18-0.37) respectively. The corresponding adjusted hazard ratios for DFS were: 0.30 (95% CI 0.18-0.50); for DSS: 0.23 (95% CI 0.15-0.36); and for OS: 0.27 (95% CI 0.18-0.39) respectively.

### HLA class I and clinical outcome in patients with HPV_DNA_+ and HPV_DNA_- OSCC

Since HPV_DNA_+ OSCC and HPV_DNA_- OSCC are regarded as two different disease entities, they have been analyzed separately[2,6–8,11,13].

In HPV_DNA_+ OSCC, absent or a weak HLA class I intensity was in general more often associated with a favorable clinical outcome than strong HLA class I intensity (Table 3). Likewise, if the fraction of positive cells was analyzed, patients with HPV_DNA_+ OSCC with low staining presented a better DFS, DSS and OS than HPV_DNA_+ patients with high staining (Table 3). Only the intensity data are presented in more detail.

**Table 3:** Univariable and multivariable analyses of HLA class I and II staining with clinical outcome in HPV DNA positive OSCC patients.

			DFS						DSS						OS					
			*Univariable*			*Multivariable^§^*			*Univariable*			*Multivariable^§^*			*Univariable*			*Multivariable^§^*		
			**HR**	**95% CI**	**p-value**	**HR**	**95% CI**	**p-value**	**HR**	**95% CI**	**p-value**	**HR**	**95% CI**	**p-value**	**HR**	**95% CI**	**p-value**	**HR**	**95% CI**	**p-value**
**HCA-2^#^**	*intensity*	strong	1.00	(ref)		1.00	(ref)		1.00	(ref)		1.00	(ref)		1.00	(ref)		1.00	(ref)	
		weak	0.43	0.17-1.1	0.087	0.42	0.16-1.1	0.082	0.40	0.14-1.2	0.090	0.40	0.14-1.2	0.089	0.50	0.22-1.1	0.097	0.46	0.20-1.1	0.068
		absent	**0.15**	**0.045-0.50**	**0.0019**	**0.17**	**0.050-0.55**	**0.003**	0.47	0.21-1.0	0.062	0.52	0.23-1.2	0.11	**0.42**	**0.21-0.85**	**0.016**	**0.46**	**0.23-0.94**	**0.033**
	*fraction*	>76%	1.00	(ref)		1.00	(ref)		1.00	(ref)		1.00	(ref)		1.00	(ref)		1.00	(ref)	
		51-75%	0.92	0.34-2.5	0.87	1.0	0.38-2.9	0.94	0.70	0.20-2.4	0.58	0.79	0.22-2.8	0.71	0.59	0.21-1.7	0.33	0.67	0.23-2.0	0.47
		26-50%	1.4	0.50-3.7	0.55	1.4	0.50-3.8	0.55	1.7	0.61-4.7	0.31	1.7	0.60-4.7	0.33	1.3	0.52-3.2	0.58	1.2	0.47-2.9	0.72
		1-25%	0.59	0.17-2.0	0.39	0.59	0.17-2.0	0.41	1.2	0.44-3.4	0.71	1.2	0.43-3.4	0.73	0.11	0.46-2.5	0.86	1.1	0.46-2.5	0.86
		absent	**0.18**	**0.052-0.60**	**0.0055**	**0.20**	**0.058-0.68**	**0.010**	0.61	0.25-1.4	0.26	0.68	0.28-1.6	0.38	0.48	0.23-1.0	0.055	0.55	0.26-1.2	0.11
**HC-10^#^**	*intensity*	strong	1.00	(ref)		1.00	(ref)		1.00	(ref)		1.00	(ref)		1.00	(ref)		1.00	(ref)	
		weak	0.59	0.26-1.4	0.22	0.58	0.25-1.4	0.21	0.71	0.32-1.6	0.39	0.70	0.32-1.6	0.39	0.56	0.27-1.2	0.12	0.52	0.25-1.1	0.083
		absent		-	-		**-**	**-**	**0.10**	**0.014-0.75**	**0.025**	**0.12**	**0.016-0.91**	**0.040**	**0.22**	**0.066-0.70**	**0.011**	**0.26**	**0.078-0.83**	**0.024**
	*fraction*	>76%	1.00	(ref)		1.00	(ref)		1.00	(ref)		1.00	(ref)		1.00	(ref)		1.00	(ref)	
		51-75%	0.70	0.24-2.0	0.52	0.66	0.22-1.9	0.45	0.89	0.34-2.3	0.81	0.83	0.32-2.2	0.71	0.73	0.31-1.8	0.48	0.68	0.28-1.6	0.38
		26-50%	2.6	0.97-6.8	0.057	2.2	0.85-6.2	0.10	2.5	0.96-6.7	0.062	2.3	0.85-6.2	0.10	1.7	0.68-4.5	0.25	1.4	0.55-3.7	0.47
		1-25%	0.59	0.14-2.5	0.47	0.54	0.13-2.3	0.41	0.60	0.14-.2.5	0.49	0.55	0.13-2.3	0.42	0.62	0.19-2.0	0.42	0.55	0.17-1.8	0.33
		absent		-	-		-	-	**0.12**	**0.016-0.86**	**0.035**	0.14	0.018-1.0	0.051	**0.24**	**0.073-0.78**	**0.018**	**0.28**	**0.084-0.91**	**0.035**
**LGII-612.14^#^**	*intensity*	strong	1.00	(ref)		1.00	(ref)		1.00	(ref)		1.00	(ref)		1.00	(ref)		1.00	(ref)	
		weak	0.31	0.041-2.3	0.25	0.39	0.051-2.9	0.32	0.62	0.14-2.7	0.52	0.83	0.19-3.7	0.40	0.73	0.22-2.4	0.086	0.92	0.27-3.1	0.89
		absent	1.4	0.71-2.9	0.32	1.4	0.71-2.9	0.36	1.3	0.67-2.7	0.41	1.3	0.67-2.7	0.81	1.7	0.93-3.0	0.61	1.6	0.91-2.9	0.099
	*fraction*	>76%	1.00	(ref)		1.00	(ref)		1.00	(ref)		1.00	(ref)		1.00	(ref)		1.00	(ref)	
		51-75%	0.83	0.24-2.9	0.77	0.86	0.25-3.0	0.82	2.5	0.76-5.6	0.16	2.1	0.77-5-6	0.15	1.6	0.68-4.0	0.27	1.7	0.68-4.0	0.27
		26-50%	0.72	0.16-3.2	0.66	0.77	0.17-3.4	0.73	0.46	0.060-3.6	0.46	0.49	0.063-3.8	0.49	0.31	0.042-2.4	0.27	0.32	0.043-2.4	0.27
		1-25%		-	-		-	-	1.3	0.36-4.6	0.71	1.3	0.36-4.6	0.71	0.88	0.26-3.0	0.83	0.83	0.24-2.9	0.77
		absent	1.3	0.61-2.7	0.50	1.3	0.60-2.7	0.53	1.6	0.73-3.5	0.24	1.6	0.72-3-5	0.26	1.7	0.91-3.3	0.092	1.7	0.87-3.2	0.13

Abbreviations: HPV human papillomavirus; OSCC, oropharyngeal squamous cell carcinoma; DFS, disease-free survival; DSS, disease-specific survival; OS, Overall survival; HR, Hazards ratio ; CI, confidence interval

§ Adjusted for sex, age, tumour stage and tumour localization

# Antibodies used to detect HLA class I and II

**Figure 1:**
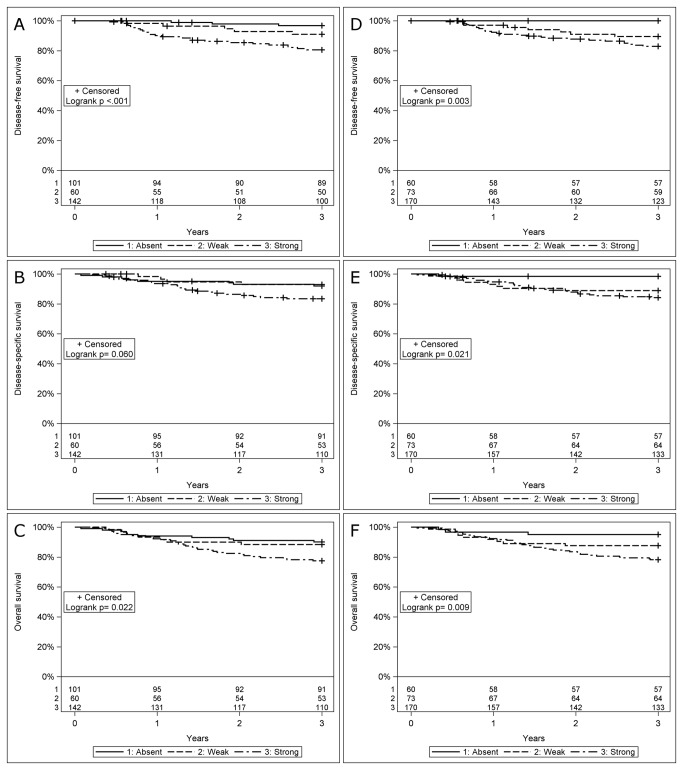
Kaplan-Meier curves for disease-free survival (DFS), disease-specific survival (DSS) and overall survival (OS) in patients with HPV positive oropharyngeal squamous cell carcinoma (OSCC) with known HLA class I expression. (A) DFS stratified for HCA-2 intensity, (B) DSS stratified for HCA-2 intensity, (C) OS stratified for HCA-2 intensity, (D) DFS stratified for HC-10 intensity, (E) DSS stratified for HC-10 intensity, and (F) OS stratified for HC-10 intensity. HPV_DNA_+ OSCC with absent HLA class I intensity had a significant better clinical outcome than tumors with strong HLA class I intensity, while weak intensity staining presented an intermediate survival (HCA-2: DFS p<0.001; DSS p=0.060; OS p=0.022; HC-10: DFS p=0.003, DSS p=0.021 and OS p=0.009, with the log-rank test). Notably, the difference observed in the HCA-2 DSS analysis did not reach significance, although the trend was similar.

In a Kaplan-Meier analysis, patients with HPV_DNA_+ OSCC with an absence of HLA class I had a better DFS, DSS and OS than those with tumors with strong HLA class I expression. Patients with HPV_DNA_+ OSCC with weak HLA class I expression presented an intermediate survival (Figure 1). 

More specifically, the 3-year DFS rates in the groups with absent, weak or strong staining for HCA-2 were 97% (95% CI 90-99); 91% (95% CI 80-96); and 81% (95% CI 73-86) respectively (Figure 1A). Corresponding numbers for DSS in the three staining categories (absent, weak and strong) were 92% (95% CI 84-96); 93% (95% CI 82-97) and 83% (95% CI 76-89) respectively (Figure 1B); and for OS 91% (95% CI 83-95); 88% (95% CI 77-94) and 77% (95% CI 70-83) respectively (Figure 1C).

A similar pattern was obtained for HC-10 staining, with 3-year DFS in the absent, weak and strong staining groups of 100%; 89% (95% CI 79-95); and 83% (95% CI 76-88) respectively (Figure 1D). Corresponding numbers for DSS in the three staining categories (absent, weak and strong) were 98% (95% CI 89-100); 89% (95% CI 79-94) and 84% (95% CI 77-89) respectively (Figure 1E), and for OS these were 95% (95% CI 85-98); 88% (95% CI 78-94) and 78% (95% CI 71-84) respectively (Figure 1F).

In a multivariate analysis, including sex, age, tumor site and stage, absence of HLA class I intensity was still a determinant of favorable clinical outcome in the HPV_DNA_+ group (Table 3). However, this was not the case when analyzing only fractions of positive cells (Table 3). 

In the HPV_DNA_- group, the opposite trend was generally observed. The absence of HLA class I staining corresponded to a worse clinical outcome (Table S2 and Figure S2). 

### HLA class *I*, treatment and clinical outcome in patients with HPV_DNA_+ OSCC

**Figure 2:**
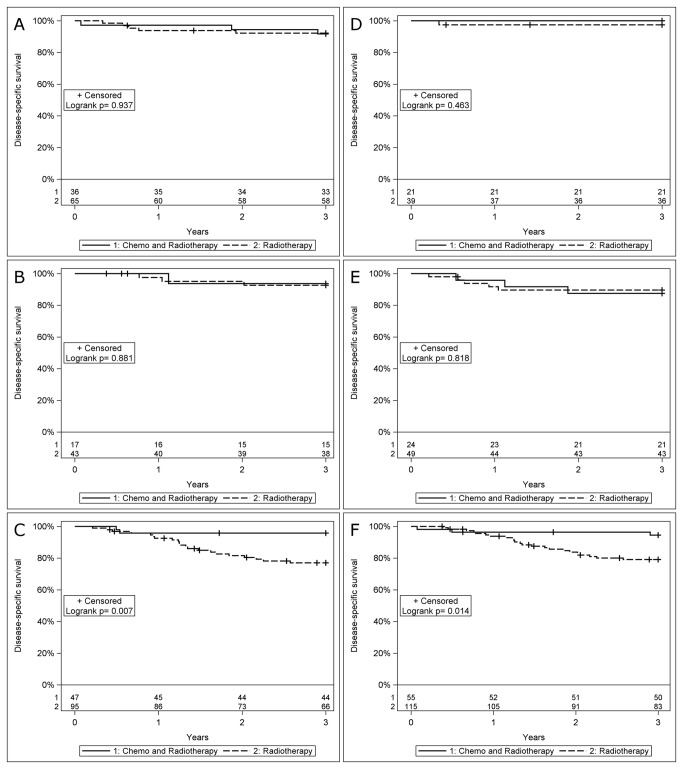
Survival presented with Kaplan-Meier curves, and analyzed using the logrank test, for disease-specific survival (DSS) in patients with HPV-positive oropharyngeal squamous cell carcinoma (HPV_DNA_+ OSCC) with known HLA class I intensity and different treatment regimes. (A) DSS in HPV_DNA_+ OSCC with absent HCA-2 intensity stratified for radiotherapy (RT) and induction chemotherapy-RT, (B) DSS in HPV_DNA_+ OSCC with weak HCA-2 intensity stratified for radiotherapy (RT) and induction chemotherapy-RT, (C) DSS in HPV_DNA_+ OSCC with strong HCA-2 intensity stratified for radiotherapy (RT) and induction chemotherapy-RT, (D) DSS in HPV_DNA_+ OSCC with absent HC-10 intensity stratified for radiotherapy (RT) and induction chemotherapy-RT, (E) DSS in HPV_DNA_+ OSCC with weak HC-10 intensity stratified for radiotherapy (RT) and induction chemotherapy-RT, (F) DSS in HPV_DNA_+ OSCC with strong HC-10 intensity stratified for radiotherapy (RT) and induction chemotherapy-RT.

The possible impact of HLA class I expression on treatment with RT *vs.* CRT was examined, although the two groups were not entirely homogenous since different RT and CRT regimens were used. Furthermore, there was most probably a selection bias for more patients with a poor clinical status receiving only RT than CRT. A Kaplan-Meier analysis was performed for DFS, DSS and OS and presented for DSS in Figure 2. 

In HPV_DNA_+ OSCC with absence of HLA class I, there were no significant differences in DFS, DSS (Figure 2A and 2D) and OS in patients treated with CRT compared to RT: HCA-2: p=0.91, p=0.94 and p= 0.68 respectively; and HC-10: p=1.00, p=0.46 and p=0.20 respectively. 

Similarly, there were no differences in DFS, DSS (Figure 2B and 2E) and OS when the same analysis was performed in HPV_DNA_+ OSCC with weak HLA class I intensity for HCA-2: p=0.15, p=0.88 and p=1.0 respectively; and HC-10: p=0.27, p=0.82 and p=0.99 respectively. 

However, patients with HPV_DNA_+ OSCC with strong HLA class I intensity had a significantly better DFS, DSS and OS if treated with CRT than with RT as shown for HCA-2: p=0.030, p=0.007 (Figure 2C), p=0.002 respectively; and HC-10: p=0.036, p=0.014 (Figure 2F) and p=0.007 respectively.

### HLA class II and clinical outcome in patients with HPV_DNA_+ and HPV_DNA_- OSCC

HLA class II expression did not influence the clinical outcome in HPV_DNA_+ OSCC (Table 3). In HPV_DNA_- OSCC strong HLA class II staining indicated a better clinical outcome (DFS: p=0.064; DSS: p=0.020; OS: p=0.004) (data not shown and Table S2). 

### p16^INK4a^, HPV_DNA_ status, HLA class I and prognosis

Overexpression of p16^INK4a^ correlated to a favorable DFS, DSS and OS irrespective of HPV status (log rank: p<0.0001 in all endpoints), and in HPV_DNA_+ OSCC (DFS: p=0.055; DSS: p<0.001; OS: p<0.001). 

**Figure 3:**
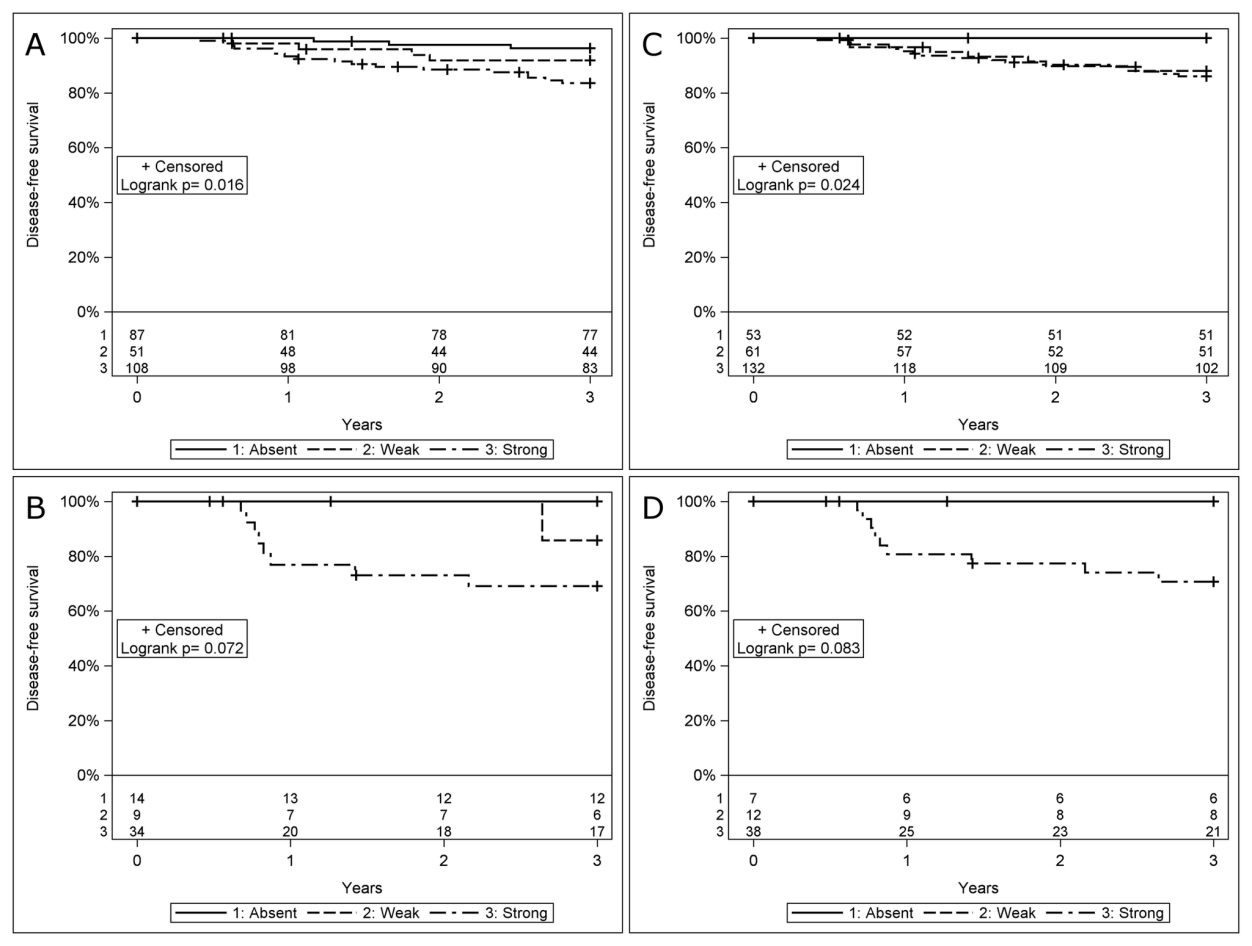
Survival presented with Kaplan-Meier curves, and analyzed using the logrank test, for disease-free survival (DFS), in patients with HPV-positive (HPV_DNA_+) and p16^INK4a^ positive/negative oropharyngeal squamous cell carcinoma (OSCC). (A) DFS in HPV_DNA_+ p16^INK4a^ positive OSCC stratified for HCA-2 intensity (p=0.016), (B) DFS in HPV_DNA_+ p16^INK4a^ negative OSCC stratified for HCA-2 intensity (p=0.072), (C) DFS in HPV_DNA_+ p16^INK4a^ positive OSCC stratified for HC-10 intensity (p=0.024), (D) DFS in HPV_DNA_+ p16^INK4a^ negative OSCC stratified for HC-10 intensity (p=0.083).

In a subgroup analysis, patients with HPV_DNA_+ OSCC with an absence of or weak HLA class I intensity staining generally presented a better clinical outcome than those with OSCC with a strong tumor HLA class I expression, irrespectively of p16^INK4a^ status. More specifically, in HPV_DNA_+ and p16^INK4a^ positive OSCC, absence of or weak HLA class I intensity was an indicator of a favorable DFS (Figure 3A and C), DSS and OS, as compared to strong HLA intensity staining. However, statistical significance was only obtained for DFS. The generally higher p-values were most likely due to an overall better survival for HPV_DNA_+ p16^INK4a^ positive OSCC with strong HLA class I intensity. 

A similar pattern was obtained for HPV_DNA_+ and p16^INK4a^ negative OSCC, with absence of or weak HLA class I tumor intensity staining being an indicator of a favorable DFS (Figure 3B and D), DSS and OS, as compared to strong HLA intensity staining. However, due to the limited number of patients statistical significance was only obtained for DSS and OS, but not in DFS. 

## Discussion

In this large cohort of OSCC patients, a significant correlation between absent/weak HLA class I expression and a very favorable clinical outcome was observed in HPV_DNA_+ OSCC, independent of treatment regime. In contrast, HPV_DNA_+ OSCC with strong HLA class I intensity presented a worse clinical outcome. HLA class II expression was not correlated to clinical outcome in patients with HPV_DNA_+ OSCC. In HPV_DNA_- OSCC, both a strong HLA class I and a strong class II expression were associated with a better clinical outcome.

The correlation between absent HLA class I expression and favorable clinical outcome in patients with HPV_DNA_+ OSCC was in line with our previous results in TSCC[13], although the underlying mechanism for the favorable outcome is still unknown. Nonetheless, as also stated previously in the pilot study [13], the very suppression of HLA expression may be due to biologically very active HPV in the tumors, where E5 and E7 are known to have the potential to downregulate HLA expression. Such tumors are most likely sensitive to RT, since no additive survival effect was observed between RT and CRT in patients with absent/weak HLA class I staining in HPV_DNA_+ OSCC. However, whether these tumors are truly more sensitive to RT, or perhaps upregulate HLA class I expression during RT, as has been shown in other malignancies[32,33], and are targeted by the immune response, are issues that need further investigation. Other explanations may include immune selection against tumors with strong initial HLA class I expression. Alternatively, these tumors could be more sensitive to NK-cells as has been shown for example for breast cancer or cervical cancer with low HLA expression [34,35]. 

Patients with HPV_DNA_+ OSCC and strong HLA class I intensity may or may not have benefited from CRT, since we assume that there was a selection bias for patients with a worse clinical condition to receive only RT. Further studies are necessary to clarify the role of CRT for this group. 

p16^INK4a^ expression was also evaluated and showed, in line with previous reports[36–39], correlation to HPV_DNA_ status and favorable clinical outcome. When patients were stratified for HPV status, overexpression of p16^INK4a^ was a prognostic marker in HPV_DNA_+ OSCC. However, whether this correlation is due to our HPV assay sensitivity or to an actual prognostic impact remains to be elucidated. 

Interestingly, in HPV_DNA_+ OSCC absence of HLA class I resulted in a very favorable clinical outcome irrespective of p16^INK4a^ overexpression. We suggest that these tumors are indeed caused by HPV, even in those lacking 16^INK4a^ overexpression, since lack of p16^INK4a^ overexpression may be caused by other means than the absence of E7 expression, such as methylation of the 16^INK4a^ promoter[40].

The correlation between strong HLA class I expression and favorable clinical outcome in HPV_DNA_- OSCC is in line with previous studies by others and ourselves in other malignancies, including HNSCC and HPV_DNA_- TSCC, and is often explained by enhanced immune recognition[13].^,^[41–43]

Upregulated HLA class II expression correlated to a favorable clinical outcome in HPV_DNA_- OSCC similar to what has been shown for some[22,24–26], but not all malignancies[23]. Furthermore, upregulation of HLA class II antigens did not correlate to absence of/weak expression of HLA class I in HPV_DNA_+ OSCC (data not shown), which could have indicated that absence of HLA class I was compensated for by immune recognition in the context of HLA class II antigens. 

The main limitation of this study is the retrospective observational design. Moreover, it is likely that there was a selection bias for patients with a poorer clinical condition to more frequently receive only RT. Nevertheless, our OSCC cohort is one of the largest analyzed, and of the patients treated with the intention to cure >90% were included. Furthermore, irrespective of treatment with CRT or RT and a possible bias in selection of treatment, patients with HPV_DNA_+ OSCC with an absence of, or weak HLA class I expression presented very high DFS, DSS and OS.

In conclusion, patients with HPV_DNA_+ OSCC and absence of HLA class I had a very high survival, independent of treatment regime. Subsequently, a prospective experimental study should be initiated to better examine absence of HLA class I expression as a marker for de-escalation of oncological treatment. 

## Supporting Information

Table S1 **Patients with oropharyngeal squamous cell carcinoma and their tumour characteristics, treated with the intention to cure with oncological treatment separated in patients with available and not available pre-treatment biopsies.**
(PDF)

Table S2 **Univariate and multivariate analyses of HLA class I and II expression with clinical outcome in patients with HPV DNA negative tumours.**
(PDF)

Figure S1 **Representative cases of HLA class I (mAb HCA-2) and p16^INK4a^ staining.** Panel A and B shows an absent staining pattern (5x and 20x respectively) and panel C shows a strong HLA class I staining (20x). Panel D shows a positive p16^INK4a^ staining.
(TIFF)

Figure S2 **Kaplan-Meier curves for disease-free survival (DFS), disease-specific survival (DSS) and overall survival (OS) in patients with HPV DNA negative oropharyngeal squamous cell carcinoma (OSCC) with known HLA class I expression.** (A) DFS stratified for HCA-2 intensity, (B) DSS stratified for HCA-2 intensity, (C) OS stratified for HCA-2 intensity, (D) DFS stratified for HC-10 intensity, (E) DSS stratified for HC-10 intensity, and (F) OS stratified for HC-10 intensity. Patients with an absent staining presented with a significant worse survival than patients with a strong staining, while patients with a weak presented an intermediate survival (HCA-2: DFS p<0.010; HC-10: DFS p<0.001 and DSS p=0.010, with the logrank test). However, the difference observed in the HCA-2 DSS, OS and HC-10 OS analyses did not reach significance, although the trend was similar (logrank test: p=0.14, p=0.22 and 0.072 respectively).
(TIFF)

## References

[1] Chaturvedi AK, Engels EA, Pfeiffer RM, Hernandez BY, Xiao W et al. (2011) Human papillomavirus and rising oropharyngeal cancer incidence in the United States. J Clin Oncol 29: 4294-4301. doi:10.1200/JCO.2011.36.4596. PubMed: 21969503. 21969503 PMC3221528

[2] Näsman A, Attner P, Hammarstedt L, Du J, Eriksson M et al. (2009) Incidence of human papillomavirus (HPV) positive tonsillar carcinoma in Stockholm, Sweden: an epidemic of viral-induced carcinoma? Int J Cancer 125: 362-366. doi:10.1002/ijc.24339. PubMed: 19330833. 19330833

[3] Blomberg M, Nielsen A, Munk C, Kjaer SK (2011) Trends in head and neck cancer incidence in Denmark, 1978-2007: focus on human papillomavirus associated sites. Int J Cancer 129: 733-741. doi:10.1002/ijc.25699. PubMed: 20878955. 20878955

[4] Braakhuis BJ, Visser O, Leemans CR (2009) Oral and oropharyngeal cancer in The Netherlands between 1989 and 2006: Increasing incidence, but not in young adults. Oral Oncol 45: e85-e89. doi:10.1016/j.oraloncology.2008.02.011. PubMed: 19457708. 19457708

[5] de Camargo Cancela M, de Souza DL, Curado MP (2012) International incidence of oropharyngeal cancer: a population-based study. Oral Oncol 48: 484-490. doi:10.1016/j.oraloncology.2011.12.013. PubMed: 22265333. 22265333

[6] Dahlstrand H, Näsman A, Romanitan M, Lindquist D, Ramqvist T et al. (2008) Human papillomavirus accounts both for increased incidence and better prognosis in tonsillar cancer. Anticancer Res 28: 1133-1138. PubMed: 18505048. 18505048

[7] Ragin CC, Taioli E (2007) Survival of squamous cell carcinoma of the head and neck in relation to human papillomavirus infection: review and meta-analysis. Int J Cancer 121: 1813-1820. doi:10.1002/ijc.22851. PubMed: 17546592. 17546592

[8] Lindquist D, Romanitan M, Hammarstedt L, Näsman A, Dahlstrand H et al. (2007) Human papillomavirus is a favourable prognostic factor in tonsillar cancer and its oncogenic role is supported by the expression of E6 and E7. Mol Oncol 1: 350-355. doi:10.1016/j.molonc.2007.08.005. PubMed: 19383307. 19383307 PMC5543872

[9] Chung CH, Schwartz DL (2012) Impact of HPV-related head and neck cancer in clinical trials: opportunity to translate scientific insight into personalized care. Otolaryngol Clin North Am 45: 795-806. doi:10.1016/j.otc.2012.04.002. PubMed: 22793853. 22793853

[10] Pryor DI, Solomon B, Porceddu SV (2011) The emerging era of personalized therapy in squamous cell carcinoma of the head and neck. Asia Pac J Clin Oncol 7: 236-251. doi:10.1111/j.1743-7563.2011.01420.x. PubMed: 21884435. 21884435

[11] Gillison ML (2004) Human papillomavirus-associated head and neck cancer is a distinct epidemiologic, clinical, and molecular entity. Semin Oncol 31: 744-754. doi:10.1053/j.seminoncol.2004.09.011. PubMed: 15599852. 15599852

[12] Lewis JS Jr. (2012) p16 Immunohistochemistry as a standalone test for risk stratification in oropharyngeal squamous cell carcinoma. Head Neck Pathol 6 Suppl 1: S75-S82. doi:10.1007/s12105-012-0336-9. PubMed: 22782226. 22782226 PMC3394161

[13] Näsman A, Andersson E, Nordfors C, Grün N, Johansson H et al. (2013) MHC class I expression in HPV positive and negative tonsillar squamous cell carcinoma in correlation to clinical outcome. Int J Cancer 132: 72-81. doi:10.1002/ijc.27635. PubMed: 22592660. 22592660

[14] Bottley G, Watherston OG, Hiew YL, Norrild B, Cook GP et al. (2008) High-risk human papillomavirus E7 expression reduces cell-surface MHC class I molecules and increases susceptibility to natural killer cells. Oncogene 27: 1794-1799. doi:10.1038/sj.onc.1210798. PubMed: 17828295. 17828295

[15] Campo MS, Graham SV, Cortese MS, Ashrafi GH, Araibi EH et al. (2010) HPV-16 E5 down-regulates expression of surface HLA class I and reduces recognition by CD8 T cells. Virology 407: 137-142. doi:10.1016/j.virol.2010.07.044. PubMed: 20813390. 20813390

[16] Li H, Ou X, Xiong J, Wang T (2006) HPV16E7 mediates HADC chromatin repression and downregulation of MHC class I genes in HPV16 tumorigenic cells through interaction with an MHC class I promoter. Biochem Biophys Res Commun 349: 1315-1321. doi:10.1016/j.bbrc.2006.08.182. PubMed: 16979588. 16979588

[17] Glew SS, Connor ME, Snijders PJ, Stanbridge CM, Buckley CH et al. (1993) HLA expression in pre-invasive cervical neoplasia in relation to human papilloma virus infection. Eur J Cancer 29A: 1963-1970. PubMed: 8280490. 8280490 10.1016/0959-8049(93)90453-m

[18] Zehbe I, Höhn H, Pilch H, Neukirch C, Freitag K et al. (2005) Differential MHC class II component expression in HPV-positive cervical cancer cells: implication for immune surveillance. Int J Cancer 117: 807-815. doi:10.1002/ijc.21226. PubMed: 15981207. 15981207

[19] Glew SS, Duggan-Keen M, Cabrera T, Stern PL (1992) HLA class II antigen expression in human papillomavirus-associated cervical cancer. Cancer Res 52: 4009-4016. PubMed: 1377602. 1377602

[20] Armstrong TD, Clements VK, Martin BK, Ting JP, Ostrand-Rosenberg S (1997) Major histocompatibility complex class II-transfected tumor cells present endogenous antigen and are potent inducers of tumor-specific immunity. Proc Natl Acad Sci U S A 94: 6886-6891. doi:10.1073/pnas.94.13.6886. PubMed: 9192661. 9192661 PMC21254

[21] Armstrong TD, Clements VK, Ostrand-Rosenberg S (1998) MHC class II-transfected tumor cells directly present antigen to tumor-specific CD4+ T lymphocytes. J Immunol 160: 661-666. PubMed: 9551900. 9551900

[22] Anichini A, Mortarini R, Nonaka D, Molla A, Vegetti C et al. (2006) Association of antigen-processing machinery and HLA antigen phenotype of melanoma cells with survival in American Joint Committee on Cancer stage III and IV melanoma patients. Cancer Res 66: 6405-6411. doi:10.1158/0008-5472.CAN-06-0854. PubMed: 16778219. 16778219

[23] van Duinen SG, Ruiter DJ, Broecker EB, van der Velde EA, Sorg C et al. (1988) Level of HLA antigens in locoregional metastases and clinical course of the disease in patients with melanoma. Cancer Res 48: 1019-1025. PubMed: 3338074. 3338074

[24] Matoba K, Iizuka N, Gondo T, Ishihara T, Yamada-Okabe H et al. (2005) Tumor HLA-DR expression linked to early intrahepatic recurrence of hepatocellular carcinoma. Int J Cancer 115: 231-240. doi:10.1002/ijc.20860. PubMed: 15688398. 15688398

[25] Momburg F, Herrmann B, Moldenhauer G, Möller P (1987) B-cell lymphomas of high-grade malignancy frequently lack HLA-DR, -DP and -DQ antigens and associated invariant chain. Int J Cancer 40: 598-603. doi:10.1002/ijc.2910400504. PubMed: 3316049. 3316049

[26] Esteban F, Ruiz-Cabello F, Concha A, Pérez-Ayala M, Sánchez-Rozas JA et al. (1990) HLA-DR expression is associated with excellent prognosis in squamous cell carcinoma of the larynx. Clin Exp Metastasis 8: 319-328. doi:10.1007/BF01810678. PubMed: 2350918. 2350918

[27] Ramqvist T, Du J, Lundén M, Ahrlund-Richter S, Ferreira J et al. (2011) Pre-vaccination prevalence of human papillomavirus types in the genital tract of 15-23-year-old women attending a youth health clinic in Stockholm, Sweden. Scand J Infect Dis 43: 115-121. doi:10.3109/00365548.2010.526957. PubMed: 20964488. 20964488

[28] Hutter H, Hammer A, Blaschitz A, Hartmann M, Ebbesen P et al. (1996) Expression of HLA class I molecules in human first trimester and term placenta trophoblast. Cell Tissue Res 286: 439-447. doi:10.1007/s004410050713. PubMed: 8929346. 8929346

[29] Perosa F, Luccarelli G, Prete M, Favoino E, Ferrone S et al. (2003) Beta 2-microglobulin-free HLA class I heavy chain epitope mimicry by monoclonal antibody HC-10-specific peptide. J Immunol 171: 1918-1926. PubMed: 12902494. 12902494 10.4049/jimmunol.171.4.1918

[30] Grandea AG 3rd, Androlewicz MJ, Athwal RS, Geraghty DE, Spies T (1995) Dependence of peptide binding by MHC class I molecules on their interaction with TAP. Science 270: 105-108. doi:10.1126/science.270.5233.105. PubMed: 7569935. 7569935

[31] Temponi M, Kekish U, Hamby CV, Nielsen H, Marboe CC et al. (1993) Characterization of anti-HLA class II monoclonal antibody LGII-612.14 reacting with formalin fixed tissues. J Immunol Methods 161: 239-256. doi:10.1016/0022-1759(93)90300-V. PubMed: 8505553. 8505553

[32] Chiriva-Internati M, Grizzi F, Pinkston J, Morrow KJ, D'Cunha N et al. (2006) Gamma-radiation upregulates MHC class I/II and ICAM-I molecules in multiple myeloma cell lines and primary tumors. In Vitro Cell Dev Biol Anim 42: 89-95. doi:10.1290/0508054.1. PubMed: 16759154. 16759154

[33] Reits EA, Hodge JW, Herberts CA, Groothuis TA, Chakraborty M et al. (2006) Radiation modulates the peptide repertoire, enhances MHC class I expression, and induces successful antitumor immunotherapy. J Exp Med 203: 1259-1271. doi:10.1084/jem.20052494. PubMed: 16636135. 16636135 PMC3212727

[34] Mehta AM, Jordanova ES, Kenter GG, Ferrone S, Fleuren GJ (2008) Association of antigen processing machinery and HLA class I defects with clinicopathological outcome in cervical carcinoma. Cancer Immunol Immunother 57: 197-206. PubMed: 17622526. 17622526 10.1007/s00262-007-0362-8 PMC2082063

[35] Madjd Z, Spendlove I, Pinder SE, Ellis IO, Durrant LG (2005) Total loss of MHC class I is an independent indicator of good prognosis in breast cancer. Int J Cancer 117: 248-255. doi:10.1002/ijc.21163. PubMed: 15900607. 15900607

[36] Kumar B, Cordell KG, Lee JS, Worden FP, Prince ME et al. (2008) EGFR, p16, HPV Titer, Bcl-xL and p53, sex, and smoking as indicators of response to therapy and survival in oropharyngeal cancer. J Clin Oncol 26: 3128-3137. doi:10.1200/JCO.2007.12.7662. PubMed: 18474878. 18474878 PMC2744895

[37] Mellin Dahlstrand H, Lindquist D, Björnestål L, Ohlsson A, Dalianis T et al. (2005) P16(INK4a) correlates to human papillomavirus presence, response to radiotherapy and clinical outcome in tonsillar carcinoma. Anticancer Res 25: 4375-4383. PubMed: 16334111. 16334111

[38] Ang KK, Harris J, Wheeler R, Weber R, Rosenthal DI et al. (2010) Human papillomavirus and survival of patients with oropharyngeal cancer. N Engl J Med 363: 24-35. doi:10.1056/NEJMoa0912217. PubMed: 20530316. 20530316 PMC2943767

[39] Marklund L, Näsman A, Ramqvist T, Dalianis T, Munck-Wikland E et al. (2012) Prevalence of human papillomavirus and survival in oropharyngeal cancer other than tonsil or base of tongue cancer. Cancer Med 1: 82-88. doi:10.1002/cam4.2. PubMed: 23342257. 23342257 PMC3544432

[40] Rocco JW, Sidransky D (2001) p16(MTS-1/CDKN2/INK4a) in cancer progression. Exp Cell Res 264: 42-55. doi:10.1006/excr.2000.5149. PubMed: 11237522. 11237522

[41] Meissner M, Reichert TE, Kunkel M, Gooding W, Whiteside TL et al. (2005) Defects in the human leukocyte antigen class I antigen processing machinery in head and neck squamous cell carcinoma: association with clinical outcome. Clin Cancer Res 11: 2552-2560. doi:10.1158/1078-0432.CCR-04-2146. PubMed: 15814633. 15814633

[42] Ogino T, Shigyo H, Ishii H, Katayama A, Miyokawa N et al. (2006) HLA class I antigen down-regulation in primary laryngeal squamous cell carcinoma lesions as a poor prognostic marker. Cancer Res 66: 9281-9289. doi:10.1158/0008-5472.CAN-06-0488. PubMed: 16982773. 16982773

[43] Andersson E, Villabona L, Bergfeldt K, Carlson JW, Ferrone S et al. (2012) Correlation of HLA-A02* genotype and HLA class I antigen down-regulation with the prognosis of epithelial ovarian cancer. Cancer Immunol Immunother 61: 1243-1253. doi:10.1007/s00262-012-1201-0. PubMed: 22258792. 22258792 PMC8693725

