# Supplementary material for: HLA Class I and II Expression in Oropharyngeal Squamous Cell Carcinoma in Relation to Tumor HPV Status and Clinical Outcome
Source: PLoS One. 2013 Oct 10;8(10):e77025. doi: 10.1371/journal.pone.0077025 (PMC3794938; doi:10.1371/journal.pone.0077025)
Supplement: Table S1 — Patients with oropharyngeal squamous cell carcinoma and their tumour characteristics, treated with the intention to cure with oncological treatment separated in patients with available and not available pre-treatment biopsies. (PDF) [file pone.0077025.s001.pdf]

Supplementary Table 1. Patients with oropharyngeal squamous cell carcinoma and their tumour characteristics, treated with the intention to cure with oncological treatment separated in patients with available and not available pre-treatment biopsies.

Patient charachteristics			OSCC patients *with* pre-treatment biopsies (N=439)		OSCC patients *without* pre-treatment biopsies (N=45)		All identified OSCC patients, treated with the intention to cure (N=484)		p value
			N	%	N	%	N	%	
**Age**	*Mean (years)*		61		61		61		ns
	Median (years)		60		61		60		
	Range (years)		30-90		40-93		30-93		
	Inter-quartile range (years)		54-67		53-69		54-68		
**Diagnosis**	*malignant neoplasm of the base of tongue (C01.9)*		103	24%	9	20%	112	23%	ns
	malignant neoplasm of the palate (C05.0-9)		22	5.0%	1	2.7%	23	4.8%	
	malignant neoplasm of the tonsil (C09.0-9)		283	65%	32	71%	315	65%	
	malignant neoplasm of the oropharynx (C10.0-9)		31	7.1%	3	6.7%	34	7.0%	
**Sex**	*female*		119	27%	11	24%	130	27%	ns
	male		320	73%	34	76%	354	73%	
**Tumour size**	*T1*		94	21%	13	29%	107	22%	ns
	T2		153	35%	14	31%	167	35%	
	T3		97	22%	7	16%	104	21%	
	T4		95	22%	11	24%	106	22%	
**Nodal disease**	*N0*		103	23%	11	24%	114	24%	ns
	N1		87	20%	9	20%	96	20%	
	N2a		60	14%	7	16%	67	14%	
	N2b		117	27%	11	24%	128	26%	
	N2c		51	12%	5	11%	56	12%	
	N3		18	4.1%	2	4.4%	20	4.0%	
	NX		3	0.68%	0	0%	3	1.0%	
**Distant metastasis**	*M0*		429	98%	44	98%	473	98%	ns
	M1		3	0.68%	0	0%	3	1.0%	
	MX		7	1.6%	1	2.2%	8	2.0%	
**Tumour Stage**	*I*		14	3.2%	2	4.4%	16	3.0%	ns
	II		36	8.2%	5	11%	41	8.0%	
	III		109	25%	9	20%	118	24%	
	IVa		251	57%	27	60%	278	57%	
	IVb		26	5.9%	2	4.4%	28	6.0%	
	IVc		3	0.68%	0	0%	3	1.0%	
**Treatment**	*Induction chemotherapy and radiation*	*conventional*	231	53%	3	7.0%	234	48%	0.007
		accelerated	72	16%	10	22%	82	17%	
	Radiation and concomittant cisplatin	*accelerated*	0	0%	0	0%	0	0%	
		conventional	0	0%	1	2.0%	1	0%	
	Radiation	*conventional*	37	8.4%	23	51%	60	12%	
		accelerated	99	23%	8	18%	107	22%	
**Brachytherapy boost**	*Not administered*		342	78%	36	80%	378	78%	ns
	Administered		97	22%	9	20%	106	22%	
**Concomittant Cetuximab**	*Not administered*		390	89%	40	89%	430	89%	ns
	Administered		49	11%	5	11%	54	11%	
**Smoking**	*Never*		112	26%	14	31%	126	26%	ns
	Former (>15 years ago)		61	14%	6	13%	67	14%	
	Former (<15 years ago)		65	15%	11	24%	76	16%	
	Current upon diagnosis		201	46%	14	31%	215	44%	
**Survived 3 years after diagnosis**	*yes*		323	74%	32	71%	355	73%	ns
	no		116	26%	13	29%	129	27%	
