# Supplementary material for: HLA Class I and II Expression in Oropharyngeal Squamous Cell Carcinoma in Relation to Tumor HPV Status and Clinical Outcome
Source: PLoS One. 2013 Oct 10;8(10):e77025. doi: 10.1371/journal.pone.0077025 (PMC3794938; doi:10.1371/journal.pone.0077025)
Supplement: Table S2 — Univariate and multivariate analyses of HLA class I and II expression with clinical outcome in patients with HPV DNA negative tumours. (PDF) [file pone.0077025.s002.pdf]

Supplementary Table 2. Univariate and multivariate analyses of HLA class I and II expression with clinical outcome in patients with HPV DNA negative tumours.

			DFS						DSS						OS					
			Univariable			*Multivariable*[§]			*Univariable*			*Multivariable*[§]			*Univariable*			*Multivariable*[§]		
			**HR**	**95% CI**	**p-value**	**HR**	**95% CI**	**p-value**	**HR**	**95% CI**	**p-value**	**HR**	**95% CI**	**p-value**	**HR**	**95% CI**	**p-value**	**HR**	**95% CI**	**p-value**
**HCA-2**[#]	*intensity*	strong	1			1			1			1			1			1		
		weak	0.64	0.25-1.6	0.35	0.65	0.26-1.7	0.37	1.5	0.79-2.7	0.22	1.5	0.81-3.3	0.17	1.4	0.81-2.4	0.23	1.4	0.80-3.4	0.24
		absent	2.4	1.1-5-2	**0.022**	2.3	1.0-5.1	**0.039**	2.0	0.97-3.9	0.060	1.6	0.78-2.7	0.24	1.7	0.90-3.2	0.11	1.5	0.74-2.7	0.31
	fraction of positive cells	>76%	1			1			1			1			1			1		
		51-75%	0.90	0.26-3.1	0.88	0.92	0.26-3.2	0.89	1.2	0.49-2.9	0.68	1.3	0.73-2.9	0.56	1.1	0.47-2.4	0.90	1.1	0.46-2.4	0.89
		26-50%	0.55	0.13-2-4	0.42	0.57	0.13-2.5	0.45	0.86	0.33-2.3	0.76	0.95	0.40-2.8	0.91	0.77	0.32-1.8	0.55	0.83	0.35-2.0	0.69
		1-25%	1.0	0.29-3.5	0.98	0.98	0.28-3.4	0.97	0.99	0.38-2.6	0.98	1.1	0.36-2.5	0.91	1.0	0-46-23	0.94	1.1	0.48-2.5	0.83
		absent	2.6	1.2-5-6	**0.017**	2.5	1.1-5.5	**0.027**	1.7	0.84-3.3	0.14	1.5	0.53-3.2	0.29	1.3	0.77-2.6	0.26	1.2	0.65-2.3	0.54
**HC-10**[#]	*intensity*	strong	1			1			1			1			1	-	-	1		
		weak	1.3	0.58-2-9	0.39	1.2	0.51-2.6	0.72	1.5	0.82-2.9	0.18	1.3	0.72-2.5	0.37	1.4	0.78-2.4	0.28	1.1	0.65-2.0	0.12
		absent	5.7	2.1-15	**0.0005**	5.1	1.8-15	**0.002**	3.3	1.4-7.6	**0.0046**	2.6	1.1-6.0	**0.029**	2.4	1.1-5.4	**0.03**	1.9	0.85-4.4	0.65
	fraction of positive cells	>76%	1			1			1			1			1			1		
		51-75%	1.3	0.48-3.4	0.62	1.2	0.44-3.3	0.73	1.0	0.42-2.4	0.99	0.87	0.36-2.1	0.76	0.71	0.31-1.7	0.44	0.57	0.24-1.3	0.20
		26-50%		-	-		-	-	0.63	0.086-4.6	0.64	0.60	0.081-4.4	0.62	0.45	0.062-3.2	0.43	0.40	0.055-2.9	0.37
		1-25%	2.2	0.65-7.3	0.20	2.0	0.55-7.1	0.30	1.7	0.59-4.7	0.34	1.2	0.40-3.5	0.76	1.2	0.43-3.3	0.75	0.78	0.27-2.2	0.64
		absent	5.7	2.1-15	**0.0006**	5.2	1.8-15	**0.002**	3.0	1.3-6.8	**0.0085**	2.3	0.99-5.4	0.054	2.1	0.95-4.7	**0.065**	1.6	0.71-3.7	0.25
**LGII-612.14**[#]	*intensity*	strong	1			1			1			1			1			1		
		weak	3.3	1.0-10	**0.042**	3.9	1.2-13	0.024	2.3	0.79-6.7	0.13	3.2	1.1-9.6	0.035	2.3	0.87-6.2	0.094	3.1	1.2-8.5	**0.025**
		absent	2.4	1.0-5.5	**0.049**	2.3	0.97-5.4	0.060	2.6	1.3-5.2	**0.0071**	2.6	1.3-5.2	**0.009**	2.8	1.5-5.3	**0.0014**	2.7	1.4-5.1	**0.002**
	fraction of positive cells	>76%	1			1			1			1			1			1		
		51-75%	1.1	0.21-5-5	0.94	1.0	0.19-5.3	0.99	1.7	0.46-6.4	0.42	1.5	0.40-5.6	0.56	1.2	0.36-4.2	0.74	1.1	0.33-3.9	0.83
		26-50%	2.1	0.51-9.0	0.30	2.0	0.47-8.4	0.35	1.4	0.26-7.0	0.72	1.2	0.24-6.3	0.82	1.0	0.20-4.7	0.97	0.94	0.19-4.5	0.93
		1-25%	1.5	0.28-7.5	0.66	1.4	0.27-7.2	0.70	2.7	0.71-9.9	0.15	2.4	0.65-0.1	0.19	2.4	0.75-7.5	0.14	2.4	0.75-7.6	0.14
		absent	2.1	0.79-5.5	0.14	2.0	0.73-5.3	0.18	3.1	1.2-7.8	**0.020**	2.7	1.1-7.0	**0.036**	2.8	1.3-6.3	**0.0099**	2.6	1.2-5.7	**0.021**

Abbreviations: HPV, human papillomavirus; OSCC, oropharyngeal squamous cell carcinoma; DFS, disease-free survival; DSS, disease-specific survival; OS, Overall survival; HR, Hazards ratio ; CI, confidence interval

[§] Adjusted for sex, age, tumour stage and tumour localization

[#] Antibodies used to detect HLA class I and II
