# Supplementary figures and images for: HLA Class I and II Expression in Oropharyngeal Squamous Cell Carcinoma in Relation to Tumor HPV Status and Clinical Outcome
Source: PLoS One. 2013 Oct 10;8(10):e77025. doi: 10.1371/journal.pone.0077025 (PMC3794938; doi:10.1371/journal.pone.0077025)

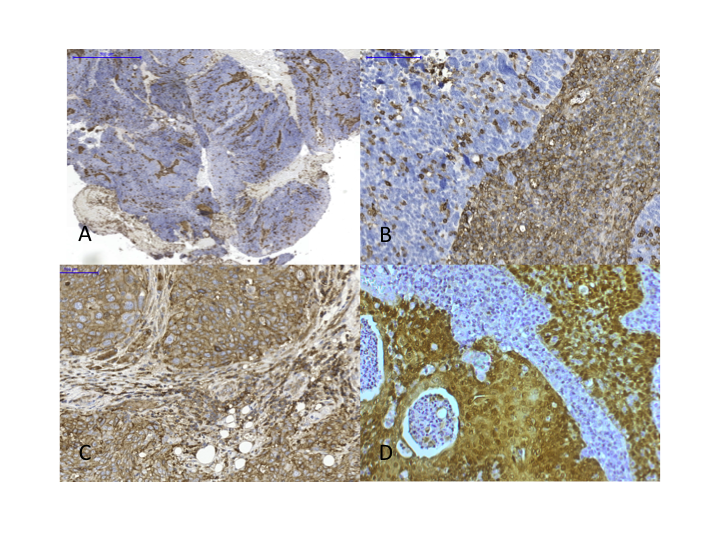

Supplement: Figure S1 — Representative cases of HLA class I (mAb HCA-2) and p16INK4a staining. Panel A and B shows an absent staining pattern (5x and 20x respectively) and panel C shows a strong HLA class I staining (20x). Panel D shows a positive p16INK4a staining. (TIFF) [file pone.0077025.s003.tiff]
